# Nucleic acids enrichment of fungal pathogens to study host-pathogen interactions

**DOI:** 10.1038/s41598-019-54608-x

**Published:** 2019-12-02

**Authors:** Antonio Rodríguez, Brecht Guillemyn, Paul Coucke, Mario Vaneechoutte

**Affiliations:** 1 Laboratory Bacteriology Research, Department of Diagnostic Sciences, Faculty of Medicine and Health Sciences, Ghent University, Ghent, 9000 Belgium; 2 Center for Medical Genetics Ghent, Ghent University Hospital, Department of Biomolecular Medicine, Ghent, 9000 Belgium

**Keywords:** Microbiology, Fungi

## Abstract

Fungal infections, ranging from superficial to life-threatening infections, represent a major public health problem that affects 25% of the worldwide population. In this context, the study of host-pathogen interactions within the host is crucial to advance antifungal therapy. However, since fungal cells are usually outnumbered by host cells, the fungal transcriptome frequently remains uncovered. We compared three different methods to selectively lyse human cells from *in vitro* mixes, composed of *Candida* cells and peripheral blood mononuclear cells. In order to prevent transcriptional modification, the mixes were stored in RNAlater. We evaluated the enrichment of fungal cells through cell counting using microscopy and aimed to further enrich fungal nucleic acids by centrifugation and by reducing contaminant nucleic acids from the host. We verified the enrichment of fungal DNA and RNA through qPCR and RT-qPCR respectively and confirmed that the resulting RNA has high integrity scores, suitable for downstream applications. The enrichment method provided here, *i.e*., lysis with Buffer RLT followed by centrifugation, may contribute to increase the proportion of nucleic acids from fungi in clinical samples, thus promoting more comprehensive analysis of fungal transcriptional profiles. Although we focused on *C. albicans*, the enrichment may be applicable to other fungal pathogens.

## Introduction

Superficial fungal infections represent a major public health problem worldwide, affecting 25% of the population. These infections are caused mainly by several species of dermatophytes of which *Trichophyton rubrum* is the most common^1^. Though superficial fungal infections are rarely life-threatening, they generally require very long and tedious antifungal treatments^2^. Mucosal infections such as vulvovaginal candidiasis are also very common, affecting millions of women every year. In fact, it is the second most common type of vaginal infection after bacterial vaginosis^3^. This infection develops into recurrent vulvovaginal candidiasis in many patients (5–8%) which impacts directly on their social lives and can be fatal in immunocompromised individuals^4,5^. Systemic, severe and life-threatening fungal infections, have also a significant impact on public health with cryptococcosis, aspergillosis and candidiasis as main diseases, with candidiasis as the most common of them^6^. They have a high rate of mortality, reaching 46% in candidaemia cases in Europe^7^. Candidiasis is mainly caused by *C. albicans*, although other *Candida* species have emerged during the last years, such as *C. glabrata*, *C. parapsilosis* and *C. tropicalis*, and very recently the multidrug-resistant pathogen *C. auris* has been involved in several outbreaks^8,9^.

Given the clinical relevance of fungal infections, there have been efforts to elucidate host-pathogen interactions in recent years, involving candidiasis^10–12^, aspergillosis^13–16^ and cryptococcosis^17,18^. However, most of these studies were focused on the transcriptome of the host. Transcriptomic studies which identify comprehensively differently expressed genes from the fungus are predominantly limited to *in vitro* experiments in which cells are cultured^19^ or animal models infected with a high inoculum of the pathogen^20^, which is usually not representative for the low amount of fungal cells encountered in the host. Although *in vitro* experiments contribute to improve our knowledge regarding the mechanisms of fungal pathogenicity and the immune response of the host, they reflect within-host interactions only to a limited extent, because these are further influenced by signalling pathway networks involving different metabolites, cell types and other microorganisms that are present in the ecosystem of the host. The use of a high burden of the pathogen in animal studies counterbalances the proportion of the fungus that is present in clinical samples. In fact, RNA content from the fungal pathogen was recently found to constitute only about 0.1% of total RNA^21^. Therefore, for true host-pathogen interaction studies, carried out on clinical samples, there is a need to enrich fungal RNA to obtain a more comprehensive analysis of transcriptional profiles from the pathogen side. To our knowledge, there are only three studies that have attempted to solve this question and developed different enrichment methods. Amorim-Vaz *et al*.^21^ used a large set of *Candida*-specific probes to selectively enrich fungal mRNAs, in combination with subsequent RNA-seq, Andes *et al*.^22^ developed a simple approach to enrich fungal RNA through the lysis of human cells with Triton X-100 and Hebecker *et al*.^23^ used buffer RLT (Qiagen) with a homogenizer to lyse murine cells, although none of these studies started from RNAlater.

In this paper, we compared three different methods to enrich fungal nucleic acids from *in vitro* mixes composed of different amounts of *Candida* cells and peripheral blood mononuclear cells (PBMCs) that had been stored in RNAlater in order to halt transcription. We evaluated the efficiency of the enrichment through cell counting, quantitative PCR (qPCR) and quantitative reverse transcription PCR (RT-qPCR). Finally, we checked RNA quality of fungal RNA obtained after the enrichment.

## Results

### Brief outline of the study

**Figure 1:**
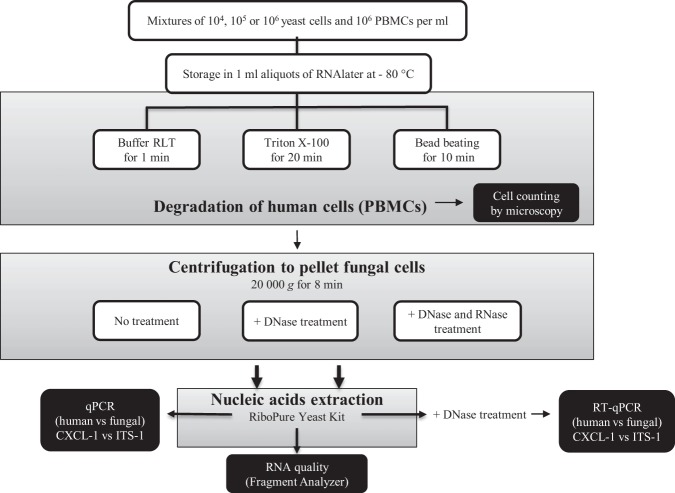
A schematic overview of the study set up.

Figure 1 summarizes the plan of the study. Three different methods to enrich fungal cells and further enrich nucleic acids from pathogenic fungi were compared. First, we prepared aliquots of mixes composed of 10^6^ yeast cells/ml and 10^6^ PBMCs/ml, and stored these mixtures in aliquots of 1 ml in RNAlater at −80 °C.

For the three different lysis treatments, we determined the number of yeast cells and PBMCs that survived the treatment by means of microscopy.

Subsequently, we also investigated whether fungal nucleic acids could be further enriched by centrifugation to precipitate intact yeast cells and to reduce human DNA and RNA from the lysed PBMCs, and we quantified DNA and RNA by qPCR and RT-qPCR respectively, and analysed RNA integrity with a Fragment Analyzer.

Finally, we determined the efficacy of these enrichment procedures for mixtures containing yeast cells reduced 10- and 100-fold relative to the human counterpart.

### Enrichment of fungal cells through lysis of human cells

**Figure 2:**
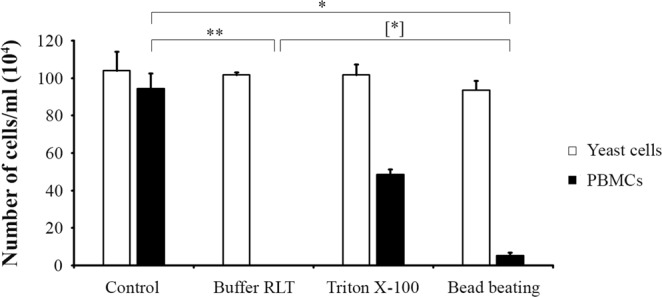
Number of yeast cells and PBMCs per ml determined by a haemocytometer, at magnification of 400x, after different cell lysis methods. Error bars represent the standard deviations of results from six biological replicates. Statistically significant differences are marked with one (*p* < 0.05) or two (*p* < 0.005) asterisks (Friedman test) and with one bracketed asterisk (*p* < 0.05) (Wilcoxon test).

Unlike fungal cells, human cells do not have a protective cell wall to deal with adverse conditions. This distinction can be used to enrich fungal cells through the differential lysis of human cells, which can potentially constitute the first step to enrich nucleic acids from the pathogenic fungus in a clinical sample. Here, we tested the effect of three different methods, all at room temperature, to lyse human cells: i) chemically with Triton X-100 for 20 min and ii) with Buffer RLT supplemented with 1% *β*-mercaptoethanol (*i.e*., 143 mM) for at least 1 min (in house protocol), and iii) mechanically by bead beating with 0.5 mm zirconium beads in saline for 10 min. Results are depicted in Fig. 2. Using microscopy, we observed that the number of yeast cells remained invariable among all three methods considered in the study.

For PBMCs, we observed that Buffer RLT completely lysed human cells (0 cells/ml) and that bead beating lysed most of them (5.3 × 10^4^ residual cells/ml). This reduction was significant as compared to the control (9.4 × 10^5^ cells/ml) (*p* < 0.05) and in addition, the differences between Buffer RLT and bead beating were significant as well. Triton X-100 did not effectively lyse human cells (4.9 × 10^5^ residual cells/ml). Thus, these results indicate that Buffer RLT and bead beating selectively lyse human cells and therefore, these methods can be used to enrich fungal cells after RNAlater storage.

### Treatment with Buffer RLT is the most efficient method to enrich fungal DNA

As we observed that Buffer RLT and bead beating in saline did reduce the number of human cells, while not affecting the number of yeast cells, we next questioned whether DNA from the fungal pathogen would be enriched as well. For this purpose, the mixes were treated with the three methods of cell lysis mentioned above, but followed by a centrifugation step (20 000 *g*, 8 min, room temperature), aiming at concentrating fungal cells and reducing nucleic acids and cell debris from human cells.

qPCR results of nucleic acids extracted with the RiboPure Yeast Kit are shown in Table 1 (human DNA and fungal DNA), targeting CXCL1 (human gene) and the internal transcribed spacer region 1 (ITS-1) DNA (yeast gene), respectively. Treatment with Buffer RLT followed by centrifugation induced a significant reduction of human DNA (*p* < 0.05), resulting in a Cq value of 35.9 which corresponds to only 0.09% human DNA as compared to the control (Cq value of 25.3) (Table 1). Enrichment with bead beating also reduced significantly human DNA (Cq value of 27.8) but a paired samples *t*-test showed that Buffer RLT is significantly more efficient (*p* < 0.001). Triton X-100 reduced human DNA to 63.8% (Cq value of 26.0), but this was not statistically significant. Additional incorporation of a DNase treatment to remove contaminating human DNA from samples treated with Buffer RLT did not further improve the enrichment, as Cq values of 36.21 and 36.50 were obtained before and after DNase treatment (see Supplementary Table S1). In addition, Table 1 shows that fungal DNA was not affected by any of the enrichment methods considered in this study. We concluded that bead beating and treatment with Buffer RLT enriched fungal cells as well as fungal DNA, by reducing the human DNA content of the mixes, with Buffer RLT treatment being the most efficient method.

**Table 1:** Effect of different enrichment methods on PCR-based amplification^a^ of human DNA, fungal DNA, human RNA and fungal RNA.

Human DNA	Cq value^b^	SD	Log cells/ml^c^	CV	DNA %^c^	CV
No enrichment	25.31	0.46	5.99	0.02	100.00	0.26
Buffer RLT	35.89**	0.59	2.96	0.06	0.09	0.32
Triton X-100	26.00	0.28	5.79	0.01	63.82	0.17
Bead beating	27.84*	1.62	5.27	0.09	18.93	0.66
**Fungal DNA**	**Cq value**	**SD**	**Log cells/ml**^**c**^	**CV**	**DNA %**^**c**^	**CV**
No enrichment	23.20	0.22	6.05	0.01	100.00	0.13
Buffer RLT	23.50	0.47	5.97	0.02	83.08	0.25
Triton X-100	23.87	0.66	5.87	0.03	66.03	0.34
Bead beating	23.60	1.62	5.94	0.07	77.91	0.63
**Human RNA**	**Cq value**^**b**^	**SD**	**Log cells/ml**^**c**^	**CV**	**RNA %**^**c**^	**CV**
No enrichment	30.17	0.37	6.13	0.02	100.00	0.20
Buffer RLT	35.00**	0.61	4.85	0.03	5.20	0.31
Triton X-100	34.53**	0.60	4.98	0.03	6.93	0.31
Bead beating	34.25**	1.05	5.05	0.06	8.20	0.48
**Fungal RNA**	**Cq value**^**b**^	**SD**	**Log cells/ml**^**c**^	**CV**	**RNA %**^**c**^	**CV**
No enrichment	20.25	0.48	5.93	0.03	100.00	0.29
Buffer RLT	20.97	0.22	5.71	0.01	59.94	0.14
Triton X-100	37.70**	0.77	0.52	0.46	0.00	0.42
Bead beating	34.02**	0.82	1.66	0.15	0.01	0.44

^a^Human nucleic acids amplified with CXCL-1 primers and fungal nucleic acids amplified with ITS-1 primers.

^b^Cq values are means of six biological replicates composed of mixes of 10^6^ PBMCs and 10^6^ *Candida* cells. Statistically significant differences are marked with one (*p* < 0.005) or two asterisks (*p* < 0.001) (Linear mixed model).

^c^Log cells/ml, DNA percentage and RNA percentage were calculated by extrapolation of Cq values with a standard curve.

SD: Standard deviation. CV: Coefficient of variation.

### Treatment with Buffer RLT additionally enriches fungal RNA

After testing the enrichment of cells and of DNA from the pathogenic fungus with three different methods, and with the addition of a centrifugation step, we evaluated whether RNA was also enriched together with DNA. Therefore, the nucleic acids obtained from the differently treated mixes after extraction by means of the RiboPure Yeast Kit, were further treated with DNase to obtain pure RNA, and quantitative reverse transcription PCR (RT-qPCR) was carried out. We observed that treatment with Buffer RLT, Triton X-100 and bead beating resulted in very low amounts of human RNA, *i.e*. 5.2, 6.9 and 8.2% respectively (Table 1). Double enzymatic pretreatment prior to the lysis of fungal cells, first with DNase and subsequently with RNase, to remove remaining human nucleic acids, did not further improve the RNA enrichment (Supplementary Table S1). Furthermore, Table 1 shows no significant differences between treatment with Buffer RLT and the untreated control for fungal ITS-1 RNA, whereas fungal RNA drastically decreased with both bead beating and Triton X-100 treatment, *i.e*. no residual RNA and 0.01% residual RNA, respectively, indicating that not only human but also fungal RNA was lost. These results suggest that only treatment with Buffer RLT can be used to enrich fungal RNA.

### Enrichment with Buffer RLT in 1:10 and 1:100 ratios

After having shown that Buffer RLT enrichment can be used to enrich nucleic acids from the fungal pathogen in samples with equal numbers of fungal and human cells, through the lysis of human cells, the enrichment procedure was tested in *Candida*/PBMC ratios of 1:10 and 1:100. To this purpose, we prepared mixtures of 10^5^ *Candida* cells/10^6^ PBMCs per ml and 10^4^ *Candida* cells/10^6^ PMBCs per ml. Cq values after qPCR of nucleic acids extracts for human and fungal DNA and after RT-qPCR of human and fungal RNA are shown in Table 2. DNA from human cells was significantly reduced to 0.65% and 0.67% of the initial number of cells (*p* < 0.05) for the 10^5^ and 10^4^ yeast cells/ml mixtures, respectively, while DNA from yeasts was (not significantly) reduced to 68.6% and 53.1%. Accordingly, human RNA significantly dropped to 4.9% and 9.3% (*p* < 0.05), while fungal cDNA was not significantly reduced to 61.5% and 34.7% for the 10^5^ and 10^4^ yeast cells/ml mixtures. Together, these results demonstrate that enrichment with Buffer RLT enriches fungal nucleic acids from mixtures with human cells, also in cases when low proportions of fungal cells are present.

**Table 2:** Effect of Buffer RLT treatment on DNA and RNA enrichment from yeast cells for mixtures composed of different ratios of PBMCs and *Candida* cells, as determined by qPCR-based amplification^a^ of human DNA and fungal DNA, and by RT-qPCR based amplification^a^ of human RNA and fungal RNA.

Human DNA	Cq value^b^	SD	Log cells/ml^c^	CV	DNA %^c^	CV
**10**^**6**^ **PBMCs** + **10**^**5**^ ***Candida*** **cells**						
No enrichment	24.39	0.67	6.08	0.03	100.00	0.34
Buffer RLT	32.52*	0.55	3.89	0.04	0.65	0.29
**10**^**6**^ **PBMCs** + **10**^**4**^ ***Candida*** **cells**						
No enrichment	24.87	0.13	5.95	0.01	100.00	0.08
Buffer RLT	32.95*	0.65	3.77	0.05	0.67	0.33
**Fungal DNA**	**Cq value**^**b**^	**SD**	**Log cells/ml**^**c**^	**CV**	**DNA %**^**c**^	**CV**
**10**^**6**^ **PBMCs** + **10**^**5**^ ***Candida*** **cells**						
No enrichment	23.74	0.59	5.02	0.03	100.00	0.32
Buffer RLT	24.31	0.58	4.85	0.03	68.56	0.32
**10**^**6**^ **PBMCs** + **10**^**4**^ ***Candida*** **cells**						
No enrichment	27.77	0.10	3.85	0.01	100.00	0.07
Buffer RLT	28.72	0.25	3.57	0.07	53.05	0.45
**Human RNA**	**Cq value**^**b**^	**SD**	**Log cells/ml**^**c**^	**CV**	**RNA %**^**c**^	**CV**
**10**^**6**^ **PBMCs** + **10**^**5**^ ***Candida*** **cells**						
No enrichment	30.65	0.88	6.09	0.04	100.00	0.45
Buffer RLT	35.15*	0.98	4.78	0.06	4.91	0.48
**10**^**6**^ **PBMCs** + **10**^**4**^ ***Candida*** **cells**						
No enrichment	30.74	1.65	4.37	0.10	100.00	0.64
Buffer RLT	34.57*	0.71	3.34	0.06	9.30	0.36
**Fungal RNA**	**Cq value**^**b**^	**SD**	**Log cells/ml**^**c**^	**CV**	**RNA %**^**c**^	**CV**
**10**^**6**^ **PBMCs** + **10**^**5**^ ***Candida*** **cells**						
No enrichment	22.51	0.52	5.06	0.03	100.00	0.28
Buffer RLT	23.26	0.46	4.84	0.03	61.47	0.26
**10**^**6**^ **PBMCs** + **10**^**4**^ ***Candida*** **cells**						
No enrichment	25.47	0.54	4.22	0.04	100.00	0.30
Buffer RLT	27.10	0.82	3.76	0.06	34.69	0.41

^a^Human nucleic acids amplified with CXCL-1 primers and fungal nucleic acids amplified with ITS-1 primers.

^b^Cq values are means of six biological replicates. Statistically significant differences are marked with one asterisk (*p* < 0.05) (paired-samples Wilcoxon test).

^c^Log cells/ml, DNA percentage and RNA percentage were calculated by extrapolation of Cq values with a standard curve.

SD: Standard deviation. CV: Coefficient of variation.

### RNA quality is suitable for transcriptomic analysis

**Figure 3:**
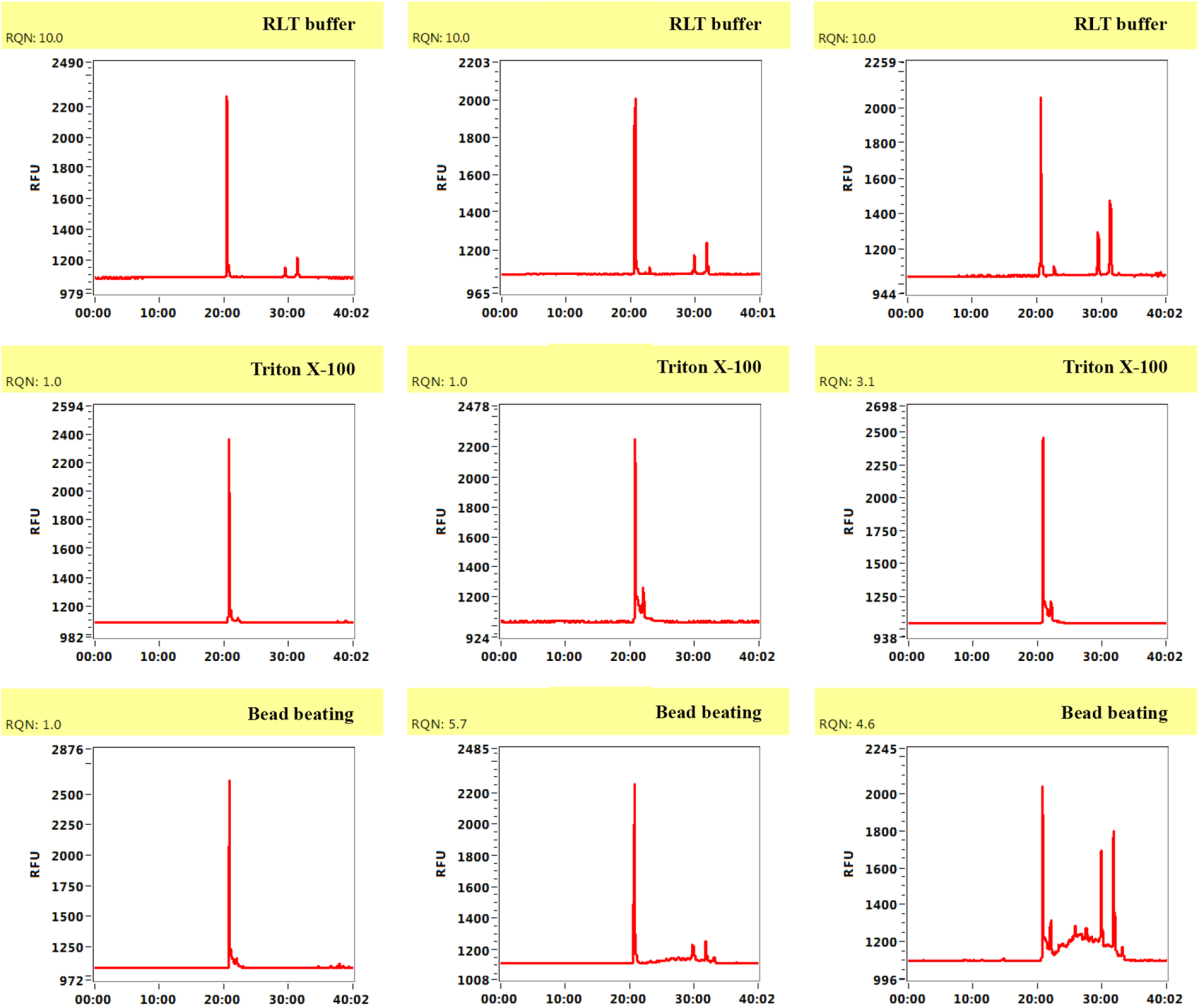
RNA integrity of some RNA samples after enrichment with buffer RLT, Triton X-100 and bead beating. Electropherogram profiles were determined with a Fragment Analyzer. Quality for Fragment Analyzer is shown as the RQN value. Reported on a scale of 1 to 10, with higher values indicating a better quality of total RNA. Values above 7 are considered to represent high quality and non-degraded RNA.

Obtaining high quality RNA is essential to perform comprehensive and reliable transcriptomic analyses. After having shown that enrichment with Buffer RLT is an efficient method to enrich both DNA and RNA from the pathogen fungus from mixes with 10- to 100-fold more human cells. We analysed RNA integrity to check to what extent it can be used for high-quality RNA-demanding experiments such as RNA-seq. Electropherograms of some RNA samples enriched with different methods are shown in Fig. 3. RQN values were around 10 for Buffer RLT enrichment and no degradation was detected. Contrarily, Triton X-100 and bead beating enrichments resulted in poor electropherogram profiles with evident RNA degradation. We concluded that enrichment with Buffer RLT confers high-quality RNA which can be used in transcriptomic analysis.

## Discussion

Host-pathogen interactions *in vivo* are frequently hampered by the low proportion of microbial cells as compared to host cells, resulting in poor transcriptional profiles from the pathogen side. In this study, we approached this problem by comparing different methods to selectively lyse human cells in order to increase the proportion of fungal cells. This enrichment of fungal cells through differential cell lysis was further complemented with centrifugation to decrease contaminant cell debris and nucleic acids of the human host cells. In our study, we started from mixtures containing 10^6^ *Candida* cells and 10^6^ PBMCs, that had been stored in RNAlater, a solution that has been shown to be as efficient as snap-frozen methods to keep transcriptomic profiles unmodified^24^. Its use is increasing during recent years because it allows short-term storage of clinical samples at above-freezing temperatures for RNA extraction purposes. This is especially relevant in clinical settings in which samples cannot be frozen immediately in liquid nitrogen.

The methods compared for differential cell lysis were Buffer RLT treatment, Triton X-100 incubation and bead beating in saline.

Triton X-100 is a non-ionic surfactant widely used to lyse different types of human cells such as red blood cells^25^, monocytes^26^ and dendritic cells^27^.

Bead beating has also been used extensively to lyse different microorganisms including bacteria^28,29^ and fungi^30,31^ and less often human cells^32^. Although bead beating is usually applied in combination with a lysis buffer, we used saline instead to avoid lysis of yeast cells.

Although Buffer RLT is a commercial lysis buffer which has been used in different protocols and kits for the lysis of cells and tissues prior to RNA extraction^33–35^, its effectiveness as a differential lysis buffer for human and fungal cells has not been studied yet.

Cell lysis was previously determined indirectly by quantification of released haemoglobin^25^, of released LDH^26^ and of metabolic activity and efficiency of protein extraction^27^. Here, we used microscopy and cell counting to directly quantify the number and type of cells that were lysed. We found that yeast cells were resistant to all three lysis methods, as we had expected, since i) the fungal cell wall confers protection against different stress conditions^36^, ii) the cell mixtures had been stored in RNAlater, which makes cells harder to lyse^37^, and iii) a combination of both bead beating and an efficient lysis buffer is needed to effectively lyse *Candida* cells^37^. We found that Buffer RLT was the most efficient method to lyse human cells. In addition, Buffer RLT was also the fastest method, since pipetting up and down several times was sufficient to lyse 100% of human cells, while Triton X-100 and bead beating required protocols lasting for 20 and 10 min respectively.

The reduced effectiveness in lysis of the PBMCs (<100%) that we obtained with Triton X-100 and bead beating in saline is probably due to the pre-storage in RNAlater, since Triton X-100 is a well-known detergent widely used in cell lysis but its use for samples stored in RNAlater has not been evaluated previously. In fact, there are no studies analysing the efficiency of cell lysis in samples stored in RNAlater apart from our recent study with *C. albicans*^37^. Buffer RLT is a more powerful chemical agent (chaotropic) that is not affected by RNAlater.

In addition, using qPCR and RT-qPCR, we demonstrated that after cell lysis with Buffer RLT, both fungal DNA and RNA can be further enriched by means of a centrifugation step that effectively reduced human nucleic acids. However, we observed that pellets of fungal cells were not strongly attached at the bottom of the tube, which may be due to the presence of large amounts of (human) DNA in the supernatant, which may increase viscosity, and interfere with the centrifugation process. Therefore, care should be taken at removal of the supernatants, to avoid removal of the pellet. For this reason, we also recommend to not include more than three samples per run of centrifugation. Using the enrichment with Buffer RLT and centrifugation, only 0.1% human RNA and 5.2% human DNA were left, which represents an important reduction of (human) nucleic acids. In a further effort to completely remove contaminating human nucleic acids, we also added DNase and RNase digestion after Buffer RLT treatment and centrifugation prior to RNA extraction. Andes *et al*.^23^, who also used Triton X-100 to enrich fungal cells, had shown that RNase digestion not only decreased human GADPH mRNA, but surprisingly also increased fungal actin mRNA, according to RT-qPCR. However, we did not succeed to reduce human RNA with Triton X-100, and enzymatic digestion did not further increase fungal RNA. Our contradictory results can be explained by the influence of RNAlater which can impair cell lysis and enzymatic treatments.

Using Buffer RLT treatment, followed by centrifugation, fungal DNA and RNA were reduced to - statistically non-significant - 83.1% and 59.9% of the original amount present, as assessed by qPCR and RT-qPCR. In summary, the Buffer RLT enrichment in combination with centrifugation was most efficient to reduce human RNA and can be performed in less than 10 min, further reducing the risk of unwanted transcriptomic profile changes.

Furthermore, we simulated the low proportion of fungal cells that is encountered in the host during colonisation or infection by reducing the number of fungal cells 10- and 100-fold relative to the number of human cells. This strategy has been used in other *in vitro* experiments^38–40^, and gives a better approximation of host-pathogen ratios present *in vivo*. It should be noted that many *in vitro* studies used a number of pathogen cells higher than that of the host cells, most likely, in order to, for example, increase the signal of luminescence during ROS production assays^41^, to increase the signal obtained during chromatographic analyses to study metabolite profiles^42^ or to stimulate a stronger immune response^43,44^. Most importantly, we obtained similar results to our experiments when using a 1:1 ratio.

Although we aimed to mimic the *in vivo* situation by mixing PBMCs and yeast cells in different ratios, a limitation of our study may be that these mixtures did not reflect possible interactions which may occur *in vivo*, such as PBMC stimulation, lysis, invasion of human cells by yeast cells or adherence between human and yeast cells.

Finally, we obtained high-quality RNA after Buffer RLT enrichment, as assessed with Fragment Analyzer, demonstrating that RNA integrity was not compromised during the enrichment process. This facilitates RNA-seq experiments and any other downstream application.

Apart from RNA, we also enriched DNA. Although we did not analyse DNA quality, we assume that Buffer RLT enrichment may be used for genomic studies as well, since DNA is more stable than RNA. Indeed, also genomic studies have to cope with the overwhelming amount of host DNA contamination in clinical samples. For example, a recent study reported about the need of DNA enrichment in metagenomic studies of sputum, and described a microbe enrichment microfluidic device coupled with DNA amplification^45^. However, although this enrichment is suitable for different bacteria, fungi are not so well recovered because of their larger size.

The enrichment method described here is based on the selective lysis of human cells, taking advantage of the protective properties that the cell wall confers to fungal cells. A comparable protocol has been published using RNAPure instead of Buffer RLT, but without explicitly showing the efficiency^46^. Andes *et al*.^23^ also enriched fungal RNA through the lysis of human cells. However, their protocol requires two incubations during 20 min at 37 °C and as such gene expression prior to RNA extraction may be changed, which would no longer match the expression profile encountered during the infection. Another study used 55.000 *Candida*-specific probes to match every gene and splicing variant of the fungal transcriptome^22^. As such, these authors carried out an enrichment after and not prior to RNA extraction. This gives an important advantage since transcriptomic profiles may change during the enrichment process. However, disadvantages of this method include certain cross-hybridization that is inherent to the technique and the cost of the probes. Using RNA-seq in two animal models, they obtained from 0.03% to 0.1% of reads aligned to *C. albicans* before the enrichment, and fungal reads were enriched to 58–69% with the probes. Since they do not provide how many of the 31–42% of unmapped reads were mapped as human, the provenance of these reads is unknown.

In summary, the clinical relevance of fungal infections has boosted the interest in host-pathogen interactions. Expanding our knowledge in this field will contribute to develop new diagnostic tools, identify potential vaccine candidates and improve the antifungal treatments we have used for more than 30 years and that are becoming increasingly obsolete^47^. The enrichment described here may enable more comprehensive analyses of transcriptional profiles from fungal pathogens within the host. Furthermore, we should evaluate whether this approach is also applicable for dermatophytes, whose cell walls are even tougher than *Candida*.

## Methods

### Culture of Candida albicans

*Candida albicans* reference strain ATCC 90028 was grown on Sabouraud Glucose Agar plates with Chloramphenicol (50 μg/ml) and transferred to Yeast extract - Peptone Dextrose broth. Cell suspensions were subcultured at 32 °C in static conditions overnight until the logarithmic growth phase was reached. Cells were counted with a microscope by using a haemocytometer (Bürker chamber). Cell suspensions were adjusted with the same broth to 2 × 10^6^, 2 × 10^5^ and 2 × 10^4^ cells/ml and centrifuged at 8000 *g* for 10 min, whereafter pellets were resuspended in 1-ml aliquots in RNAlater (Invitrogen, Carlsbad, CA) and placed at 4 °C following immediate preparation of yeast cells/PBMCs mixtures.

### PBMC isolation

PBMCs were isolated from a buffycoat as described previously^48^ after informed consent from all participants. Cell pellets were resuspended in RNAlater to a concentration of 2 × 10^6^ cells/ml and divided into 1-ml aliquots and placed at 4 °C following immediate preparation of yeast cells/PBMCs mixtures. We confirm that all methods were carried out in accordance to relevant guidelines and regulations and that all experimental protocols were approved by the ethical committee of the University of Ghent (EC/2016/0192).

### Preparation of yeast cells/PBMCs mixtures and RNAlater treatment

Volumes of 500 µl of 1 ml-aliquots containing 2 × 10^6^, 2 × 10^5^ and 2 × 10^4^ yeast cells/ml and 500 µl of 1 ml-aliquots with 2 × 10^6^ PBMCs/ml were mixed together to obtain 1-ml aliquots of 10^6^:10^6^, 10^5^:10^6^ and 10^4^:10^6^ yeasts cells:PBMCs mixtures. These mixtures were stored overnight at 4 °C to enable the RNAlater to penetrate into the cells, and subsequently stored at −80 °C.

### Cell lysis of human cells

Mixtures of yeast cells and PBMCs that had been stored at −80 °C, were thawed, centrifuged for 10 min at 20 000 *g* and resuspended in 1-ml aliquots of i) Triton X-100 (final concentration, 1%), ii) Buffer RLT (Qiagen, Hilden, Germany) supplemented with 1% *β*-mercaptoethanol and iii) saline. Buffer RLT and Triton X-100 samples were incubated for at least 1 min and 20 min respectively, while saline samples were transferred to prefilled tubes with 0.5 mm zirconium beads and further bead beaten for 10 min in a hands-free vortex genie-2 (MO BIO Laboratories, Carlsbad, CA), fitted with a vortex adapter that allows bead beating in a horizontal position.

### Microscopic visualization and cell viability determination

After the different cell lysis treatments, cell suspensions were centrifuged for 10 min at 8000 *g* to collect intact yeast cells and PBMCs. They were then resuspended in 1 ml of saline prior to cell counting with a microscope. Ten µl of this saline cell suspension was loaded into a haemocytometer. Yeast cells and PBMCs could be observed with light microscopy and were counted in 25 small squares with 0.2 mm sides (together representing a 0.1 µl volume) at a magnification of 400x. The resulting number of cells/ml was calculated as follows: cells/ml = cell number (in 25 small squares) × 1 (dilution) × 10^4^.

### Centrifugation conditions for enrichment

Centrifugation at 20 000 *g* for 8 min at room temperature was used to pellet fungal cells. Supernatants containing cell debris from human cells that had been lysed with the three different methods were discarded very carefully, since fungal pellets were very loose and they can be detached very quickly. No more than three samples were centrifuged per run of centrifugation to minimize the risk of fungal pellets being removed together with cell debris from human cells.

### DNase and RNase treatment

Pellets of intact yeast cells were treated to digest contaminating human DNA and RNA. First, pellets were resuspended in 86 µl nuclease-free water, and DNase treatment was performed by addition of 10 µl of 10x DNase I buffer and 4 µl of DNase I (2 U/µl) and by incubation for 30 min at 37 °C. Second, the yeast pellet was processed for RNase treatment by addition of 98 µl of nuclease-free water and 2 µl of RNase A Solution (4 mg/ml). The mixture was then incubated for 1 h at 37 °C.

### Nucleic acids extraction

Nucleic acids were isolated using the RiboPure Yeast Kit (Invitrogen) and following the manufacturer’s instruction of the kit. Half of the resulting nucleic acids was used directly for DNA quantification by means of qPCR, and the other half was treated with DNase I to obtain pure RNA. RNA was then analyzed for quality with a NanoDrop spectrophotometer and a Fragment Analyzer (DNF-472 High-Sensitivity Total RNA, Applied Biosystems) and subsequently quantified with RT-qPCR.

### qPCR and RT-qPCR analysis

Reverse transcription was performed for 5 min at 25 °C followed by 60 min at 42 °C with random hexamer primers and with 5 µl of total RNA according to the instructions of the manufacturer of the RevertAid First Strand cDNA Synthesis Kit (Thermo Fisher Scientific, Waltham, MA). qPCR from both cDNA and DNA was performed in duplicate with a LightCycler 480 (Roche). We used CXCL1_F forward (5′-GGA AAG AGA GAC ACA GCT GCA-3′) and CXCL1_R reverse (5′-AGA AGA CTT CTC CTA AGC GAT GC-3′) primers, previously described (Van Belleghem *et al*.^48^), targeting the human gene encoding the cytokine CXCL1, and CA_rRNA F (5′-TTT GCT TGA AAG ACG GTA-3′) and CA_rRNA R (5′-TTG AAG ATA TAC GTG GTG G-3′) targeting the ITS-1 gene of *C. albicans* (adapted from^49^). PCR master mixes to quantify both human cDNA/DNA and yeast cDNA/DNA were prepared similarly, *i.e*. Roche LC480 high resolution melting (HRM) mix (Roche, Basel, Switzerland), 0.5 µM primers and 2 µl sample (cDNA or DNA) in a final volume of 10 µl, with exception of MgCl_2_ which was used at a concentration of 2 mM and 3 mM to amplify human cDNA/DNA and yeast cDNA/DNA respectively. The thermal cycling program consisted of a pre-incubation step for 10 min at 95 °C, amplification for 45 cycles of 30 s at 95 °C, 30 s at 59 °C and 30 s at 72 °C. Results were analyzed with the LightCycler 480 software 1.5 (Roche).

### Statistical analysis

For statistical comparisons of cell lysis and PCR data (cq values), independent experiments performed with six replicates were considered. Data were analyzed using the linear mixed model for repeated measures followed by Bonferroni’s multiple testing correction, and with Friedman’s test and Wilcoxon’s test where the assumption of normality was not acceptable, using the IBM SPSS Statistics software v 25.0 (IBM, Armonk, NY, USA).

## Supplementary information

Suppl. Table S1

## Data Availability

The datasets used and analysed during the current study are available from the corresponding author on reasonable request.
