# Supplementary material for: Nucleic acids enrichment of fungal pathogens to study host-pathogen interactions
Source: Sci Rep. 2019 Dec 2;9:18037. doi: 10.1038/s41598-019-54608-x (PMC6889467; doi:10.1038/s41598-019-54608-x)
Supplement: Supplementary file 1 — Suppl. Table S1 [file 41598_2019_54608_MOESM1_ESM.pdf]

Supplementary Information

Nucleic acids enrichment of fungal pathogens to study host-pathogen interactions

Antonio Rodríguez, Brecht Guillemyn, Paul Coucke and Mario Vaneechoutte

Supplementary Table S1. Effect of DNase/RNase treatment, on quantification, expressed as Cq values[a], of human nucleic acids (extracted by means of the RiboPure Yeast Kit) of PBMC:*Candida* mixtures, pre-treated with Buffer RLT.

**Cell lysis method**	**Treatment of NA extract**	**qPCR**	**RT-qPCR**
Buffer RLT	None	36.21 (0.56)	34.13 (0.21)
Buffer RLT	DNase	36.50 (0.59)	
Buffer RLT	DNase + RNase		34.61 (1.01)

a: Cq values are means (standard deviations) of six biological replicates, obtained after qPCR and RT-qPCR with CXCL1 primers targeting human DNA and RNA. No statistically significant differences ($p > 0.05$) were observed (paired samples Wilcoxon test).
